# Acoustic and non-acoustic factors in modeling listener-specific performance of sagittal-plane sound localization

**DOI:** 10.3389/fpsyg.2014.00319

**Published:** 2014-04-23

**Authors:** Piotr Majdak, Robert Baumgartner, Bernhard Laback

**Affiliations:** Psychoacoustics and Experimental Audiology, Acoustics Research Institute, Austrian Academy of SciencesWien, Austria

**Keywords:** sound localization, localization model, sagittal plane, listener-specific factors, head-related transfer functions

## Abstract

The ability of sound-source localization in sagittal planes (along the top-down and front-back dimension) varies considerably across listeners. The directional acoustic spectral features, described by head-related transfer functions (HRTFs), also vary considerably across listeners, a consequence of the listener-specific shape of the ears. It is not clear whether the differences in localization ability result from differences in the encoding of directional information provided by the HRTFs, i.e., an acoustic factor, or from differences in auditory processing of those cues (e.g., spectral-shape sensitivity), i.e., non-acoustic factors. We addressed this issue by analyzing the listener-specific localization ability in terms of localization performance. Directional responses to spatially distributed broadband stimuli from 18 listeners were used. A model of sagittal-plane localization was fit individually for each listener by considering the actual localization performance, the listener-specific HRTFs representing the acoustic factor, and an uncertainty parameter representing the non-acoustic factors. The model was configured to simulate the condition of complete calibration of the listener to the tested HRTFs. Listener-specifically calibrated model predictions yielded correlations of, on average, 0.93 with the actual localization performance. Then, the model parameters representing the acoustic and non-acoustic factors were systematically permuted across the listener group. While the permutation of HRTFs affected the localization performance, the permutation of listener-specific uncertainty had a substantially larger impact. Our findings suggest that across-listener variability in sagittal-plane localization ability is only marginally determined by the acoustic factor, i.e., the quality of directional cues found in typical human HRTFs. Rather, the non-acoustic factors, supposed to represent the listeners' efficiency in processing directional cues, appear to be important.

## 1. Introduction

Human listeners use monaural spectral cues to localize sound sources in sagittal planes (e.g., Wightman and Kistler, 1997; van Wanrooij and van Opstal, 2005). This includes the ability to assign the vertical position of the source (e.g., Vliegen and van Opstal, 2004) and to distinguish between front and back (e.g., Zhang and Hartmann, 2010). Spectral cues are caused by the acoustic filtering of the torso, head, and pinna, and can be described by means of head-related transfer functions (HRTFs; e.g., Møller et al., 1995). The direction-dependent components of the HRTFs are described by directional transfer functions (DTFs, Middlebrooks, 1999b).

The ability to localize sound sources in sagittal planes, usually tested in psychoacoustic experiments as localization performance, varies largely across listeners (Middlebrooks, 1999a; Rakerd et al., 1999; Zhang and Hartmann, 2010). A factor contributing to the variability across listeners might be the listeners' morphology. The ear shape varies across the human population (Algazi et al., 2001) and these differences cause the DTF features to vary across individuals (Wightman and Kistler, 1997). One might expect that different DTF sets provide different amounts of cues available for the localization of a sound. When listening with DTFs of other listeners, the performance might be different, an effect we refer to in this study as the *acoustic factor* in sound localization.

The strong effect of training on localization performance (Majdak et al., 2010, Figure 7) indicates that in addition to the acoustic factor, also other listener-specific factors are involved. For example, a link between the listener-specific sensitivity to the spectral envelope shape and the listener-specific localization performance has been recently shown (Andéol et al., 2013). However, other factors like the ability to perform the experimental task, the attention paid to the relevant cues, or the accuracy in responding might contribute as well. In the present study, we consolidate all those factors to a single factor which we refer to as the *non-acoustic factor*.

In this study, we are interested in the contribution of the acoustic and non-acoustic factors to sound localization performance. As for the acoustic factor, its effect on localization performance has already been investigated in many studies (e.g., Wightman and Kistler, 1997; Middlebrooks, 1999a; Langendijk and Bronkhorst, 2002). However, most of those studies investigated *ad-hoc* listening with modified DTFs without any re-calibration of the spectral-to-spatial mapping in the auditory system (Hofman et al., 1998). By testing the *ad-hoc* localization performance to modified DTFs, two factors were simultaneously varied: the directional cues in the incoming sound, and their mismatch to the familiarized (calibrated) mapping. The acoustic factor of interest in our study, however, considers changes in the DTFs of the *own* ears, i.e., changes of DTFs without any mismatch between the incoming sound and the calibrated mapping. A localization experiment testing such a condition would need to minimize the mismatch by achieving a re-calibration. Such a re-calibration is indeed achievable in an extensive training with modified DTFs, however, the experimental effort is rather demanding and requires weeks of exposure to the modified cues (Hofman and van Opstal, 1998; Majdak et al., 2013). Note that such a long-term re-calibration is usually attributed to perceptual adaptation, in contrast to the short-term learning found to take place within hours (Zahorik et al., 2006; Parseihian and Katz, 2012).

Using a model of the localization process, the condition of a complete re-calibration can be more easily achieved. Thus, our study is based on predictions from a model of sagittal-plane sound localization (Baumgartner et al., 2013). This model assumes that listeners create an internal template set of their specific DTFs as a result of a learning process (Hofman et al., 1998; van Wanrooij and van Opstal, 2005). The more similar the representation of the incoming sound compared to a template, the larger the assumed probability of responding at the polar angle corresponding to that template (Langendijk and Bronkhorst, 2002). The model from Baumgartner et al. (2013) uses a method to compute localization performance based on probabilistic predictions and considers both acoustic factors in terms of the listener-specific DTFs and non-acoustic factors in terms of an uncertainty parameter *U*. In Baumgartner et al. (2013), the model has been validated under various conditions for broadband stationary sounds. In that model, the role of the acoustic factor can be investigated by simultaneously modifying DTFs of both the incoming sound and the template sets. This configuration allows to predict sound localization performance when listening with others' ears following a complete re-calibration to the tested DTFs.

In the following, we briefly describe the model and revisit the listener-specific calibration of the model. Then, the effect of the uncertainty representing the non-acoustic factor, and the effect of the DTF set representing the acoustic factor, are investigated. Finally, the relative contributions of the two factors are compared.

## 2. Materials and methods

### 2.1. Model

In this study, we used the model proposed by Baumgartner et al. (2013). The model relies on a comparison between an internal representation of the incoming sound and an internal template set (Zakarauskas and Cynader, 1993; Hofman and van Opstal, 1998; Langendijk and Bronkhorst, 2002; Baumgartner et al., 2013). The internal template set is assumed to be created by means of learning the correspondence between the spectral features and the direction of an acoustic event based on feedback from other modalities (Hofman et al., 1998; van Wanrooij and van Opstal, 2005). The model is implemented in the Auditory Modeling Toolbox as baumgartner2013 (Søndergaard and Majdak, 2013).

**Figure 1:**
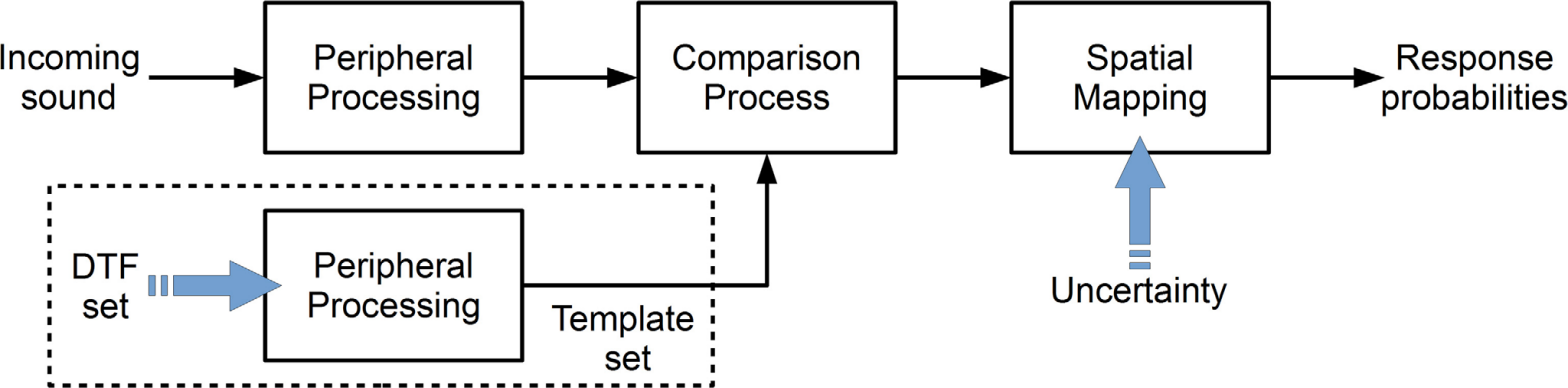
**Structure of the sound localization model from Baumgartner et al. (2013).** The incoming target sound is peripherally processed and the result is compared to an internal template set. The comparison result is mapped yielding the probability for responding at a given polar angle. The blue arrows indicate the free parameters of the corresponding sections. In the model, the DTF set and the uncertainty represent the acoustic and non-acoustic factors, respectively.

Figure 1 shows the basic structure of the model from Baumgartner et al. (2013). Each block represents a processing stage of the auditory system in a functional way. The target sound is processed in order to obtain an internal (neural) representation. This target representation is compared to an equivalently processed internal template set consisting of the DTF representations for the given sagittal plane. This comparison process is the basis of a spectral-to-spatial mapping, which yields the prediction probability for responding at a given polar angle.

In general, in this study, we used the model configured as suggested in Baumgartner et al. (2013). In the following, we summarize the model stages and their configuration, focusing on the acoustic and non-acoustic factors in the localization process.

#### 2.1.1. Peripheral processing

In the model, the same peripheral processing is considered for the incoming sound and the template. The peripheral processing stage aims at modeling the effect of human physiology while focusing on directional cues. The effect of the torso, head and pinna are considered by filtering the incoming sound by a DTF. The effect of the cochlear filtering was considered as linear Gammatone filter bank (Patterson et al., 1988). The filter bank produces a signal for each frequency band. 28 frequency bands were considered in the model, determined by the lowest frequency of 0.7 kHz, the highest frequency of 18 kHz, and the frequency spacing of the bands corresponding to one equivalent rectangular bandwidth (Glasberg and Moore, 1990). In the model, the output of each frequency band is half-wave rectified and low-pass filtered (2nd-order Butterworth filter, cut-off frequency of 1 kHz) in order to simulate the effect of the inner hair cells (Dau et al., 1996). The filtered outputs are then temporally averaged in terms of root-mean-square (RMS) amplitude, resulting in the internal representation of the sound.

#### 2.1.2. Comparison stage

In the comparison stage, the internal representation of the incoming sound is compared with the internal template set. Each template is selected by a polar angle denoted as template angle. A distance metric is calculated as a function of the template angle and is interpreted as a descriptor contributing to the prediction of the listener's response.

In the model, the distance metric is represented by the standard deviation (SD) of the inter-spectral differences between the internal representation of the incoming sound and a template calculated across frequency bands. The SD of inter-spectral differences is robust against changes in overall level and has been shown to be superior to other metrics like the inter-spectral cross-correlation coefficient (Langendijk and Bronkhorst, 2002).

#### 2.1.3. Spatial mapping

In the model, a probabilistic approach is used for the mapping of the distance metric to the predicted response probability. For a particular target angle, response angle, and ear, the distance metric is mapped by a Gaussian function to a similarity index (SI), interpreted as a measure reflecting the response probability for a response angle.

The mapping function actually reflects the *non-acoustic factor* of the localization process. In the model, the width of the Gaussian function was considered as a property of an individual listener. Baumgartner et al. (2013) assumed that a listener being more precise in the response to the same sound would need a more narrow mapping than a less precise listener. Thus, the width of the mapping function was interpreted as a listener-specific uncertainty, *U*. In the model, it accounted for listener-specific localization performance and was a free parameter in the calibration process. In Langendijk and Bronkhorst (2002), the uncertainty parameter has actually also been used (their *S*), however, it was considered to be constant for all listeners, thus representing a rather general property of the auditory system. The impact of the uncertainty *U*, representing the non-acoustic factor responsible for the listener variability on the predicted localization performance is described in the following sections.

In the model, the contribution of the two ears was considered by applying a binaural weighting function (Morimoto, 2001; Macpherson and Sabin, 2007), which reduces the contribution of the contralateral ear with increasing lateral angle of the target sound. The binaural weighting function is applied to each monaural SI, and the sum of the weighted monaural SIs yields the binaural SI.

In the model, for a given target angle, the binaural SIs are calculated as a function of the response angle, i.e., for all templates. The SI as a function of response angle is scaled to a sum of one in order to be interpreted as a probability mass vector (PMV), i.e., a discrete version of a probability density function. Such a PMV describes the listener's response probability as a function of the response angle for a given incoming sound.

### 2.2. Experimental conditions for calibration

In Baumgartner et al. (2013), the model was calibrated to the actual performance of a pool of listeners for the so-called baseline condition, for which actual data (DTFs and localization responses) were collected in two studies, namely in Goupell et al. (2010) and Majdak et al. (2013). In both studies, localization responses were collected using virtual stimuli presented via headphones. While localization performance seems to be better when using free-field stimuli presented via loudspeakers (Middlebrooks, 1999b), we used virtual stimuli in order to better control for cues like head movements, loudspeaker equalization, or room reflections. In this section, we summarize the methods used to obtain the baseline conditions in those two studies.

#### 2.2.1. Subjects

In total, 18 listeners were considered for the calibration. Eight listeners were from Goupell et al. (2010) and 13 listeners were from Majdak et al. (2013), i.e., three listeners participated in both studies. None of them had indications of hearing disorders. All of them had thresholds of 20-dB hearing level or lower at frequencies from 0.125 to 12.5 kHz.

#### 2.2.2. HRTFs and DTFs

In both Goupell et al. (2010) and Majdak et al. (2013), HRTFs were measured individually for each listener. The DTFs were then calculated from the HRTFs. Both HRTFs and DTFs are part of the ARI HRTF database (Majdak et al., 2010).

Twenty-two loudspeakers (custom-made boxes with VIFA 10 BGS as drivers) were mounted on a vertical circular arc at fixed elevations from −30° to 80°, with a 10° spacing between 70° and 80° and 5° spacing elsewhere. The listener was seated in the center point of the circular arc on a computer-controlled rotating chair. The distance between the center point and each speaker was 1.2 m. Microphones (Sennheiser KE-4-211-2) were inserted into the listener's ear canals and their output signals were directly recorded via amplifiers (FP-MP1, RDL) by the digital audio interface.

A 1729-ms exponential frequency sweep from 0.05 to 20 kHz was used to measure each HRTF. To speed up the measurement, for each azimuth, the multiple exponential sweep method was used (Majdak et al., 2007). At an elevation of 0°, the HRTFs were measured with a horizontal spacing of 2.5° within the range of ±45° and 5° otherwise. With this rule, the measurement positions for other elevations were distributed with a constant spatial angle, i.e., the horizontal angular spacing increased with the elevation. In total, HRTFs for 1550 positions within the full 360° horizontal span were measured for each listener. The measurement procedure lasted for approximately 20 min. The acoustic influence of the equipment was removed by equalizing the HRTFs with the transfer functions of the equipment. The equipment transfer functions were derived from reference measurements in which the microphones were placed at the center point of the circular arc and the measurements were performed for all loudspeakers.

The DTFs (Middlebrooks, 1999b) were calculated. The magnitude of the common transfer function (CTF) was calculated by averaging the log-amplitude spectra of all HRTFs for each individual listener and ear. The phase spectrum of the CTF was set to the minimum phase corresponding to the amplitude spectrum. The DTFs were the result of filtering HRTFs with the inverse complex CTF. Finally, the impulse responses of all DTFs were windowed with an asymmetric Tukey window (fade in of 0.5 ms and fade out of 1 ms) to a 5.33-ms duration.

#### 2.2.3. Stimulus

In Majdak et al. (2013), the experiments were performed for targets in the lateral range of ±60°. In Goupell et al. (2010), the experiments were performed for targets in the lateral range of ±10°. The direction of a target is described by the polar angle ranging from −30° (front, below eye-level) to 210° (rear, below eye-level).

The audio stimuli were Gaussian white noise bursts with a duration of 500 ms, which were filtered with the listener-specific DTFs corresponding to the tested condition. The level of the stimuli was 50 dB above the individually measured absolute detection threshold for that stimulus, estimated in a manual up-down procedure for a frontal eye-leveled position. In the experiments, the stimulus level was randomly roved for each trial within the range of ±5 dB in order to reduce the possibility of using overall level cues for localization.

#### 2.2.4. Apparatus

In both studies, Goupell et al. (2010) and Majdak et al. (2013), the virtual acoustic stimuli were presented via headphones (HD 580, Sennheiser) in a semi-anechoic room. Stimuli were generated using a computer and output via a digital audio interface (ADI-8, RME) with a 48-kHz sampling rate. A virtual visual environment was presented via a head-mounted display (3-Scope, Trivisio). It provided two screens with a field of view of 32° x 24° (horizontal x vertical dimension). The virtual visual environment was presented binocularly with the same picture for both eyes. A tracking sensor (Flock of Birds, Ascension), mounted on the top of the listener's head, captured the position and orientation of the head in real time. A second tracking sensor was mounted on a manual pointer. The tracking data were used for the 3-D graphic rendering and response acquisition. More details about the apparatus are provided in Majdak et al. (2010).

#### 2.2.5. Procedure

For the calibration, the data were collected in two studies using the same procedure. In Goupell et al. (2010), the data were the last 300 trials collected within the acoustic training, see their Sec. II. D. In Majdak et al. (2013), the data were the 300 trials collected within the acoustic test performed at the beginning of the pre-training experiments, see their Sec. II. D. In the following, we summarize the procedure used in the two studies.

In both studies, the listeners were immersed in a spherical virtual visual environment (for more details see Majdak et al., 2010). They were standing on a platform and held a pointer in their right hand. The projection of the pointer direction on the sphere's surface, calculated based on the position and orientation of the tracker sensors, was visualized and recorded as the perceived target position. The pointer was visualized whenever it was in the listeners' field of view.

Prior to the acoustic tests, listeners participated in a visual training procedure with the goal to train them to point accurately to the target. The visual training was a simplified game in the first-person perspective in which listeners had to find a visual target, point at it, and click a button within a limited time period. This training was continued until 95% of the targets were found with an RMS angular error smaller than 2°. This performance was reached within a few hundred trials.

In the acoustic experiments, at the beginning of each trial, the listeners were asked to align themselves with the reference position, keep the head direction constant, and click a button. Then, the stimulus was presented. The listeners were asked to point to the perceived stimulus location and click the button again. Then, a visual target in the form of a red rotating cube was shown at the position of the acoustic target. In cases where the target was outside of the field of view, arrows pointed towards its position. The listeners were asked to find the target, point at it, and click the button. At this point in the procedure, the listeners had both heard the acoustic target and seen the visualization of its position. To stress the link between visual and acoustic location, the listeners were asked to return to the reference position and listen to the same acoustic target once more. The visual feedback was intended to trigger a procedural training in order to improve the localization performance within the first few hundred of trials (Majdak et al., 2010). During this second acoustic presentation, the visual target remained visualized in the visual environment. Then, while the target was still visualized, the listeners had to point at the target and click the button again. An experimental block consisted of 50 targets and lasted for approximately 15 min.

### 2.3. Data analysis

In the psychoacoustic experiments, the errors were calculated by subtracting the target angles from the response angles. We separated our data analysis into confusions between the hemifields and the local performance within the correct hemifield. The rate of confusions was represented by the quadrant error (QE), which is the percentage of responses where the absolute polar error exceeded 90° (Middlebrooks, 1999b). In order to quantify the local performance in the polar dimension, the local polar RMS error (PE) was calculated, i.e., the RMS of the polar errors calculated for the data without QEs.

The listener-specific results from both Goupell et al. (2010) and Majdak et al. (2013) were pooled. Only responses within the lateral range of ±30° were considered because (1) most of the localization responses were given in that range, (2) Baumgartner et al. (2013) evaluated the model using only that range, and (3) recent evaluations indicate that predictions for that range seem to be slightly more accurate than those for more lateral ranges (Baumgartner et al., 2014). For the considered data, the average QE was 9.3% ± 6.0% and the average PE was 34° ± 5°. This is similar to the results from Middlebrooks (1999b) who tested 14 listeners in virtual condition using DTFs. His average QE was 7.7% ± 8.0% and the average PE was 29° ± 5°.

In the model, targets in the lateral range of ±30° were considered in order to match the lateral range of the actual targets from the localization experiments. For each listener, PMVs were calculated for three lateral segments with a lateral width of 20° each, and these PMVs were evaluated corresponding to the actual lateral target angles. The QE was the sum of the corresponding PMV entries outside the local polar range for which the response-target distance exceeded 90°. The PE was the discrete expectancy value within the local polar range. Both errors were calculated as the arithmetic averages across all polar target angles considered.

## 3. Results and discussion

### 3.1. Model calibration

In Baumgartner et al. (2013), the model was calibrated individually for each listener by finding the uncertainty *U* providing the smallest residual in the predictions as compared to the actual performance obtained in the localization experiments.

**Figure 2:**
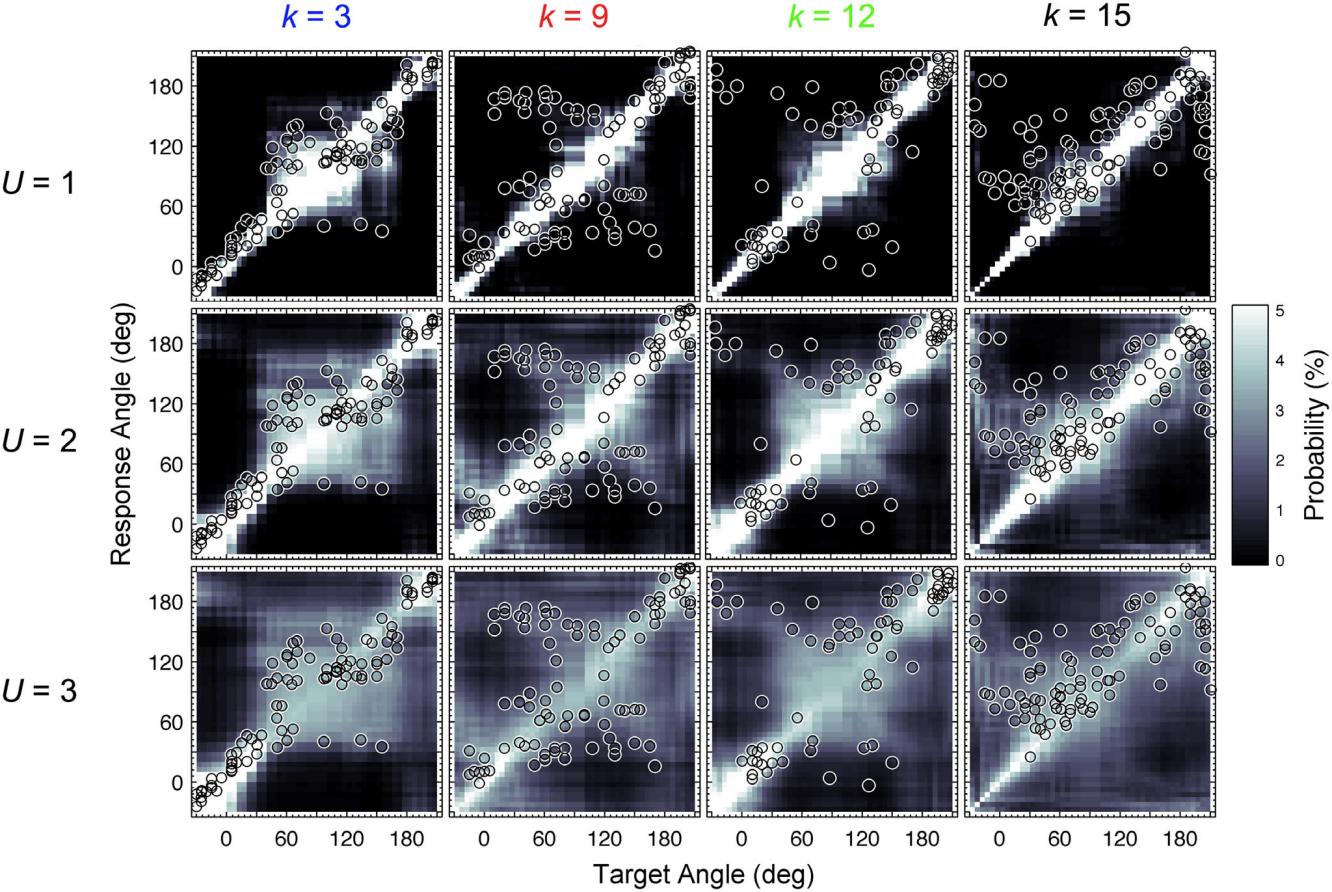
**Actual and modeled localization.** Actual localization responses (circles) and modeled response probabilities (PMVs, brightness encoded) calculated for three uncertainties *U* and four exemplary listeners indexed by *k*.

In our study, this calibration process was revisited. For each listener and all target directions, PMVs were calculated for varying uncertainty *U* ranging from 0.1 to 4.0 in steps of 0.1. Listener-specific DTFs were used for both the template set and incoming sound. Figure 2 shows PMVs and the actual localization responses for four exemplary listeners and exemplary uncertainties.

**Figure 3:**
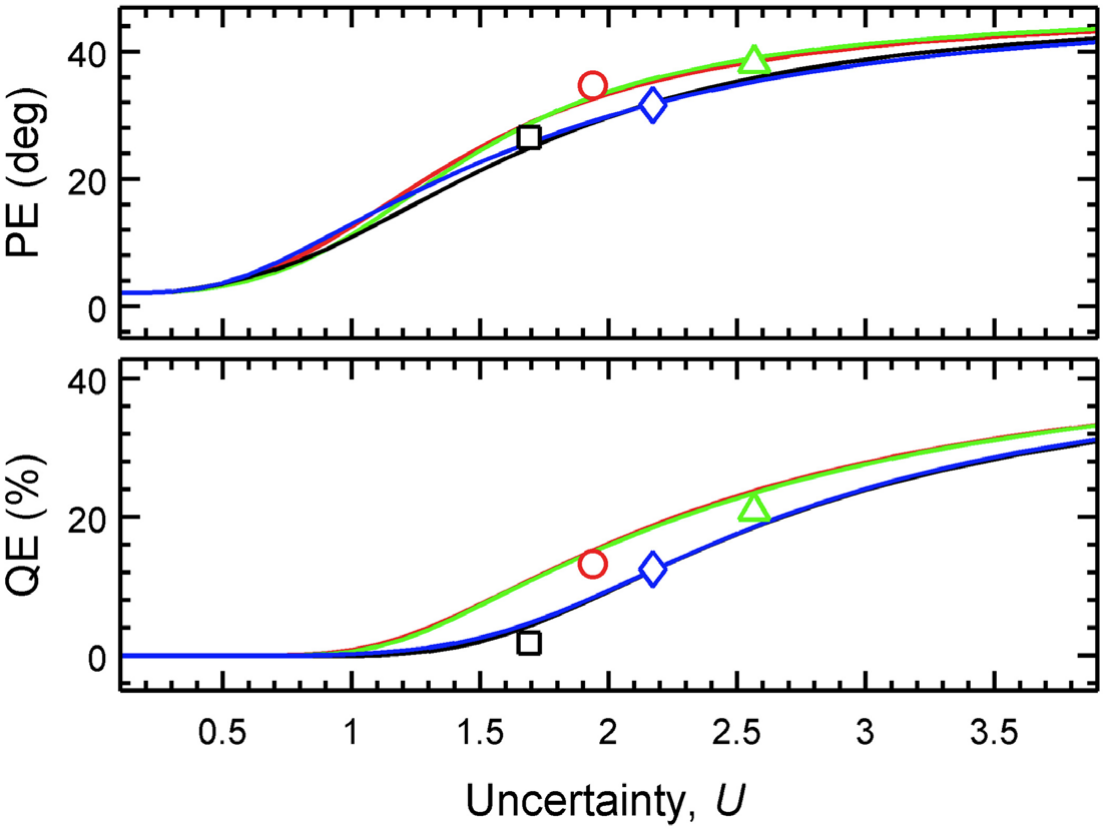
**Predicted localization performance depends on the uncertainty.** PEs and QEs are shown as functions of *U* for four exemplary listeners (*k* = 3: blue squares, *k* = 9: red triangles, *k* = 12: green diamonds, *k* = 15: black circles). Lines show the model predictions. Symbols show the actual performance obtained in the localization experiment (placement on the abscissa corresponds to the optimal listener-specific uncertainty *U_k_*).

For each listener, the predicted PEs and QEs were calculated from the PMVs, and the actual PEs and QEs were calculated from the experimental results. Figure 3 shows the predicted QEs and PEs as a function of the uncertainty for the four exemplary listeners. The symbols show the actual QEs and PEs.

In Baumgartner et al. (2013), the uncertainty yielding the smallest squared sum of residues between the actual and predicted performances (PE and QE) was considered as optimal. Using the same procedure, the optimal uncertainties *U_k_* were calculated for each listener *k* and are shown in Table 1. For the listener group, the average listener-specific uncertainty amounted to 2.05 (*SD* = 0.37).

**Table 1:** **Uncertainty *U*_k_ of individual listener with index *k***.

***k***	**1**	**2**	**3**	**4**	**5**	**6**	**7**	**8**	**9**	**10**	**11**	**12**	**13**	**14**	**15**	**16**	**17**	**18**
*x*	58	53	12	42	46	43	15	21	22	71	55	64	72	68	33	39	62	41
*U*	1.48	1.63	1.68	1.74	1.75	1.83	1.85	1.91	1.94	2.01	2.12	2.22	2.24	2.29	2.33	2.35	2.47	3.05

Listener indexed by k is identified in the ARI HRTF database by NH*x*_k_. The listeners are sorted by *k* corresponding to ascending *U_k_*.

**Figure 4:**
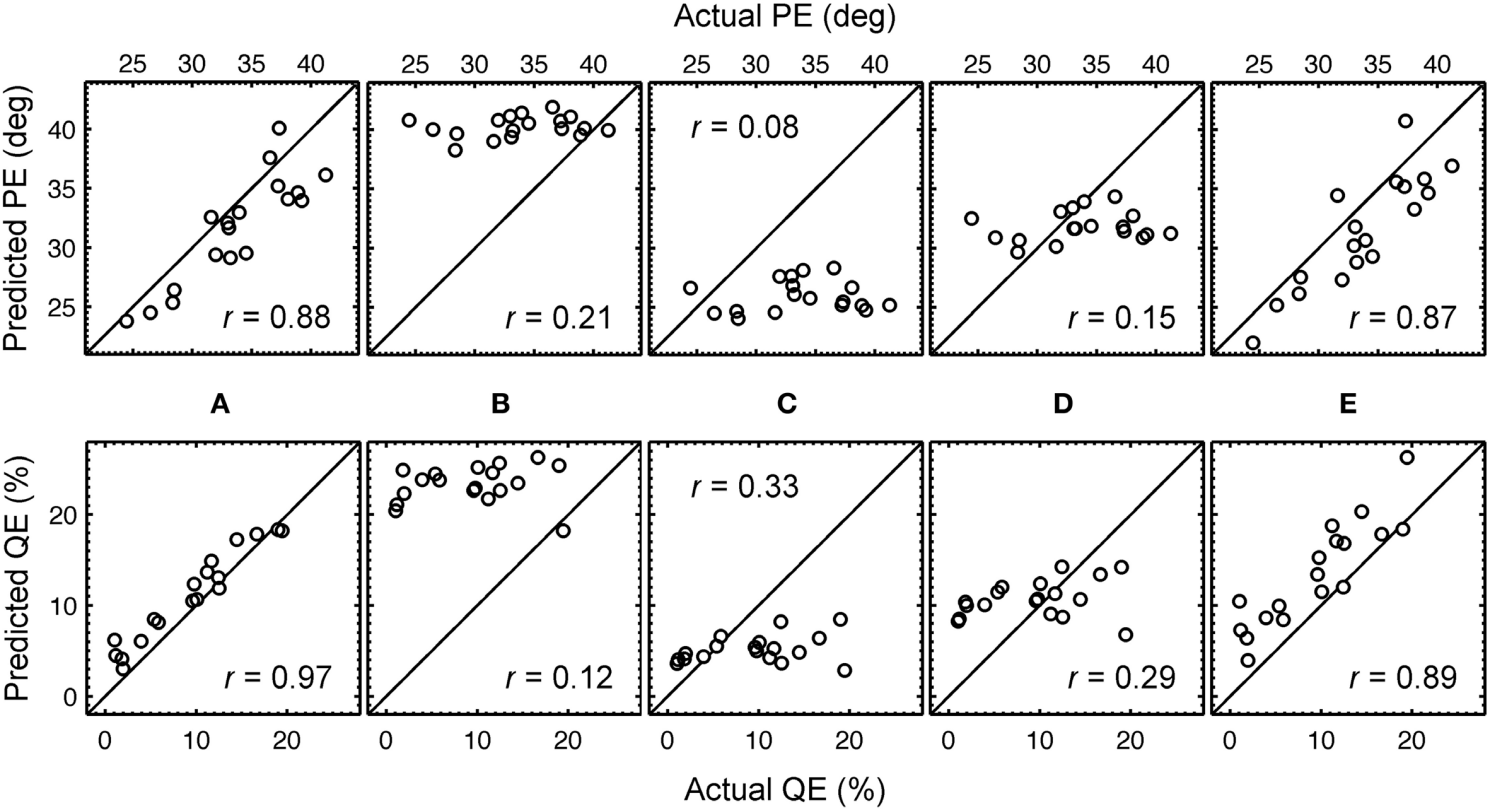
**Predicted versus actual localization performance.** Predicted PEs and QEs are shown as functions of the actual PEs and QEs, respectively, for each listener. **(A)** Optimal listener-specific uncertainties *U_k_*. **(B)** Listener-constant uncertainty yielding best correlation for PE, *U* = 2.89. **(C)** Listener-constant uncertainty yielding best correlation for QE, *U* = 1.87. **(D)** Listener-constant uncertainty from (Langendijk and Bronkhorst, 2002), *U* = 2.0. **(E)** Listener-specific uncertainties *U_k_* and the same DTF set (*k* = 14) for all listeners (see Section 3.3 for more details). The correlation coefficient is denoted by *r*.

With the optimal listener-specific uncertainties from Table 1, predictions were compared to the actual localization performances. Figure 4A shows the correspondence between the actual and predicted QEs and PEs of all listeners when using those listener-specific uncertainties. For the listener group, the correlation coefficient between actual and predicted localization errors was 0.88 for PE and 0.97 for QE. In Baumgartner et al. (2013), the model calibrated with those optimal uncertainties was evaluated in further conditions involving DTF modifications yielding correlation coefficients in the range of 0.75.

### 3.2. Non-acoustic factor: listener-specific uncertainty

In Baumgartner et al. (2013), the optimal listener-specific uncertainties were assumed to yield most accurate performance predictions. In Langendijk and Bronkhorst (2002), the effect of spectral cues was modeled by using a parameter corresponding to our uncertainty. Interestingly, that parameter was constant for all listeners and the impact of this listener-specific uncertainty is not clarified yet. Thus, in this section, we investigate the effect of uncertainty being listener-specific as compared to uncertainty being constant for all listeners, when using the model from Baumgartner et al. (2013).

Predictions were calculated with a model calibrated to uncertainty being constant for all listeners. Three uncertainties were used: (1) *U* = 2.89, which yielded largest correlation with the actual PEs of the listeners, (2) *U* = 1.87, which yielded largest correlation with the actual QEs, and (3) *U* = 2.0, which corresponds to that used in Langendijk and Bronkhorst (2002). The DTFs used for the incoming sound and the template set were still listener-specific, representing the condition of listening with own ears. The predictions are shown in Figures 4B–D. The corresponding correlation coefficients are shown as insets in the corresponding panels. From this comparison and the comparison to that for listener-specific uncertainties (Figure 4A), it is evident that listener-specific calibration is required to account for the listener-specific actual performance.

Our findings are consistent with the results from Langendijk and Bronkhorst (2002) who used a constant calibration for all listeners. The focus of that study was to investigate the change in predictions caused by the variation of spectral cues. Thus, prediction changes for different conditions *within* an individual listener were important, which, in the light of the model from Baumgartner et al. (2013), correspond to the variation of the DTFs used for the incoming sound and not to the variation of the uncertainty. *U* = 2.0 seems to be indeed an adequate choice for predictions for an “average listener”. This is supported by the similar average uncertainty of our listener group (*U* = 2.05). It is further supported by the performance predicted with *U* = 2.0, which was similar to the actual group performance. For acurate listener-specific predictions, however, listener-specific uncertainty is required.

The listener-constant uncertainty seems to have largely reduced the predicted performance variability in the listener group. In order to quantify this observation, the group SDs were calculated for predictions with listener-constant *U* from 1.1 to 2.9 in steps of 0.1 for each listener. For PE, the group SD was 0.96° ± 0.32°. For QE, the group SD was 1.34% ± 0.87%. For comparison, the group SD for predictions with listener-*specific* uncertainties was 4.58° and 5.07% for PE and QE, respectively, i.e., three times larger than those for predictions with the listener-constant uncertainties.

In summary, the listener-specific uncertainty seems to be vital to obtain accurate predictions of the listeners' actual performance. The listener-constant uncertainty drastically reduced the correlation between the predicted and actual performance. Further, the listener-constant uncertainty reduced the group variability in the predictions. Thus, as the only parameter varied in the model, the uncertainty seems to determine to a large degree the baseline performance predicted by the model. It can be interpreted as a parameter calibrating the model in order to represent a good or bad localizer; the smaller the uncertainty, the better the listeners' performance in a localization task. Notably, uncertainty is not associated with any acoustic information considered in the model, and thus, it represents the non-acoustic factor in modeling sound localization.

### 3.3. Acoustic factor: listener-specific directional cues

In the previous section, the model predictions were calculated for listeners' own DTFs in both the template set and the incoming sound; a condition corresponding to listening with own ears. With the DTFs of other listeners but own uncertainty, their performance might have been different.

For the investigation of that effect, one possibility would be to vary the quality of the DTF sets along a continuum simultaneously in both the incoming sound and the template set, and analyze the corresponding changes in the predictions. Such an investigation would be, in principle, similar to that from the previous section where the uncertainty was varied and the predicted performance was analyzed. While *U* represents a measure of the uncertainty, a similar metric would be required in order to quantify the quality differences between two DTF sets. Finding an appropriate metric is challenging. A potentially useful metric is the spectral SD of inter-spectral differences (Middlebrooks, 1999b; Langendijk and Bronkhorst, 2002) as used in the model from (Baumgartner et al., 2013) as the distance metric and thus as basis for the predictions. Being a part of the model, however, this metric is barred from being an independent factor in our investigation.

In order to analyze the DTF set variation as a parameter without any need for quantification of the variation, we systematically replaced the listeners' own DTFs by DTFs from other listeners from this study. The permutation of the DTF sets and uncertainties within the same listener group allowed us to estimate the effect of directional cues relative to the effect of uncertainty on the localization performance of our group.

**Figure 5:**
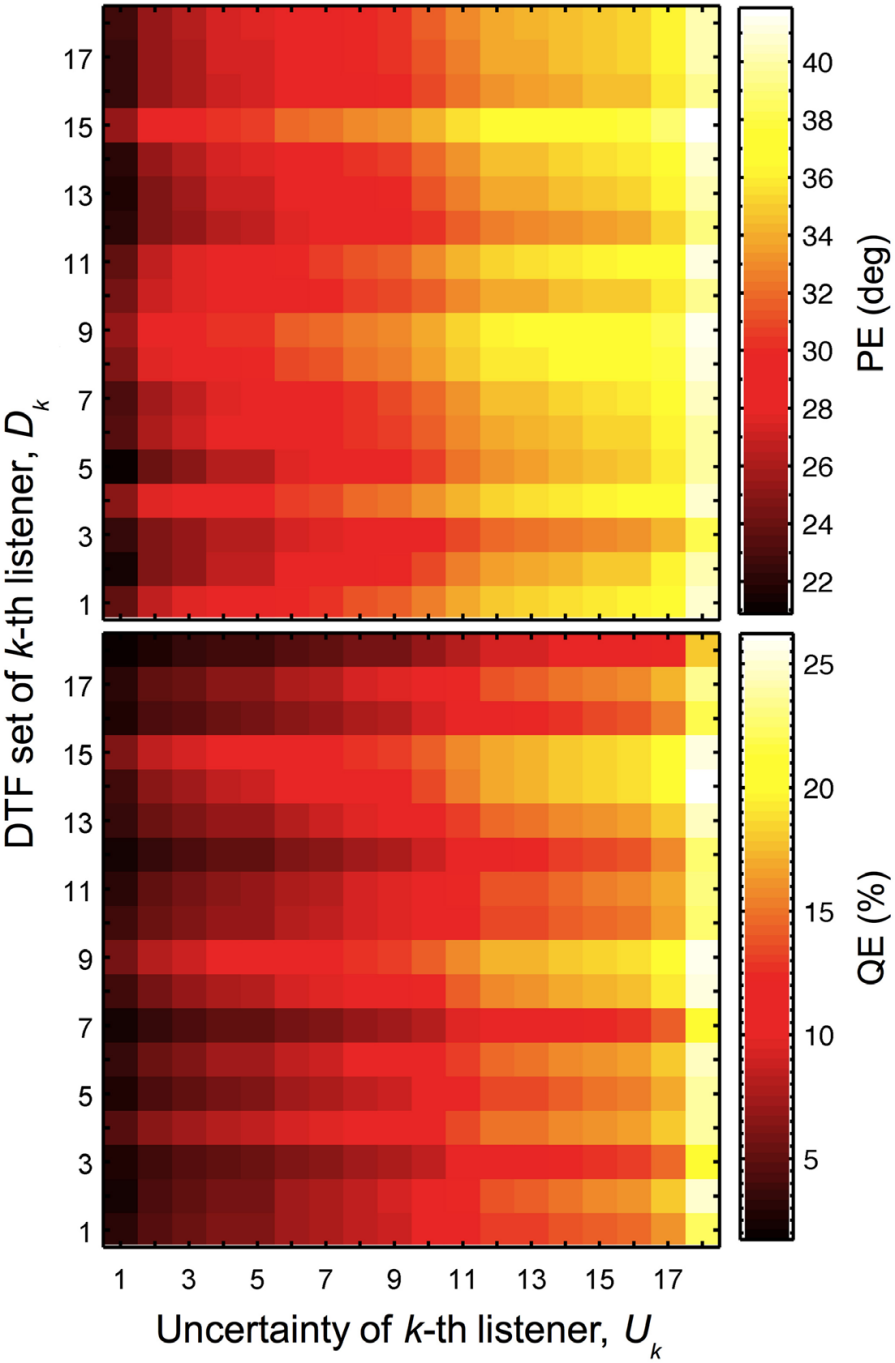
**Localization performance depends on the uncertainty and DTF set.** Predicted PEs and QEs as functions of the uncertainty of *k*-th listener (*U_k_*) and DTF set of *k*-th listener (*D_k_*).

For each listener, the model predictions were calculated using a combination of DTF sets and uncertainties of all listeners from the group. Indexing each listener by *k*, predicted PEs and QEs as functions of *U_k_* and *D_k_* were obtained, with *U_k_* and *D_k_* being the uncertainty and the DTF set, respectively, of the *k*-th listener. Figure 5 shows the predicted PEs and QEs for all combinations of *U_k_* and *D_k_*. The listener group was sorted such that the uncertainty increases with increasing *k* and the same sorting order was used for *D_k_*. This sorting order corresponds to that from Table 1.

The results reflect some of the effects described in the previous sections. The main diagonal represents the special case of identical *k* for *D_k_* and *U_k_*, corresponding to listener-specific performance, i.e., predictions for each listener's actual DTFs and optimal listener-specific uncertainty from the calibrated model described in Section 3.1. Each row, i.e., constant *D_k_* but varying *U_k_*, represents the listener-specific effect of the uncertainty described in Section 3.2, i.e., listening with own ears but having different uncertainties.

**Figure 6:**
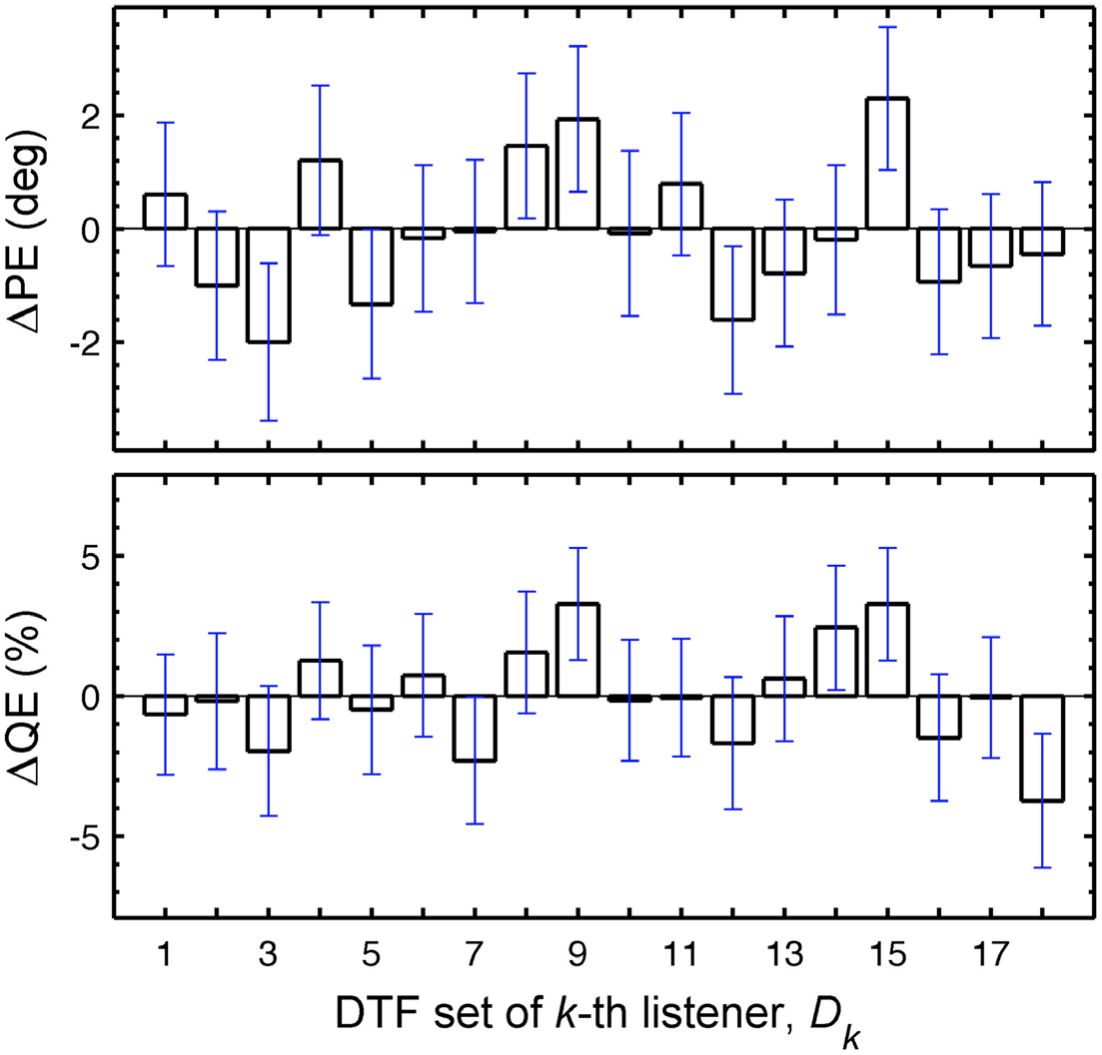
**Listener-specific performance depends on the DTF set used in the model.** ΔPEs and ΔQEs averaged over all *U_k_*s as a function of *D_k_*. ΔPEs and ΔQEs are the PEs and QEs relative to the listener-specific PEs and QEs, respectively. The whiskers show ±1 SD.

In this section, we focus on the results in the columns. Each column describes results for a constant *U_k_* but varying *D_k_*, representing the listener-specific effect of the DTF set. While the predictions show a variation across both columns and rows, i.e., substantial effects of both uncertainty and DTF set, some DTF sets show clear differences to others across all uncertainties. This analysis is, however, confounded by the different baseline performance of each listener and can be improved by considering the performance relative to the listener-specific performance. Figure 6 shows ΔPEs and ΔQEs, i.e., PEs and QEs relative to the listener-specific PEs and QEs, respectively, averaged over all uncertainties for each DTF set *D_k_*. Positive values represent the performance amount by which our listener group would deteriorate when listening with the DTF set of *k*-th listener (and being fully re-calibrated). For example, the DTF sets of listeners *k* = 9 and *k* = 15 show such deteriorations. Those DTF sets seem to have provided less accessible directional cues. Further, DTF sets improving the performance for the listeners can be identified, see for example, the DTF sets of listeners *k* = 3 and *k* = 12. These DTF sets seem to have provided more accessible directional cues. The effect of those four DTF sets can be also examined in Figure 2 by comparing the predictions for constant uncertainties, i.e., across rows.

Thus, variation of the DTF sets had an effect on the predictions suggesting that it also affects the comparison of the predictions with the actual performance. This leads to the question to what extend a constant DTF set across all listeners can explain the actual performances? It might even be the case that listener-specific DTFs are not required for accurate predictions. Thus, similarly to the analysis from Section 3.2 where the impact of listener-specific uncertainty was related to that of a listener-constant uncertainty, here, we compare the impact of listener-specific DTF sets relative to that of a listener-constant DTF set. To this end, predictions were calculated with a model calibrated to the same DTF set for all listeners but with a listener-specific uncertainty. All DTF sets from the pool of available listeners were tested. For each of the DTF sets, correlation coefficients between the actual and predicted performances were calculated. The correlation coefficients averaged over all DTF sets were 0.86 (*SD* = 0.007) for PE and 0.89 (*SD* = 0.006) for QE. Note the extremely small variability across the different DTF sets, indicating only little impact of the DTF set on the predictions. The DTF set from listener *k* = 14 yielded the largest correlation coefficients, which were 0.87 for PE and 0.89 for QE. The corresponding predictions as functions of the actual performance are shown in Figure 4E. Note the similarity to the predictions for the listener-specific DTF sets (Figure 4A). These findings have a practical implication when modeling the baseline performance of sound localization: for an arbitrary listener, the DTFs of another arbitrary listener, e.g., NH68 (*k* = 14), might still yield listener-specific predictions.

Recall that in our investigation, both the incoming sound and the template set were filtered by the same DTF set, corresponding to a condition where the listener is completely re-calibrated to those DTFs. The highest correlation found for NH68's DTF set does not imply that this DTF set is optimal for *ad-hoc* listening.

In summary, the predicted localization performance varied by a small amount depending on the directional cues provided by the different DTF sets, even when the listener-specific uncertainty was considered. Note that full re-calibration was simulated. This finding indicates that some of the DTF sets provide better access to directional cues than others. Even though the acoustic factor might contribute to the variability in localization performance across listeners, the same DTF set of a single listener (here, NH68) for modeling performance of all listeners yielded still a good prediction accuracy.

### 3.4. Relative contributions of acoustic and non-acoustic factors

Both the DTF set and the uncertainty had an effect on the predicted localization performance. However, a listener-constant DTF set provided still acceptable predictions, while a listener-constant uncertainty did not. In this section, we aim at directly comparing the relative contributions of the two factors to localization performance. To this end, we compare the SDs in the predictions as a function of each of the factors. The factor causing more variation in the predictions is assumed to have more impact on sound localization.

**Figure 7:**
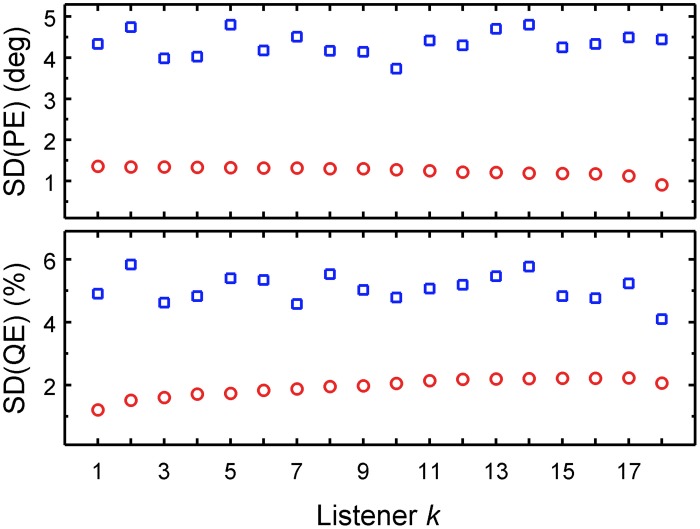
**DTF set contributes less than uncertainty to the performance variability of the group.** PE SDs and QE SDs as functions of either listener-constant DTF set calculated for listener-specific uncertainties (*U_k_* varied, blue squares) or the listener-constant uncertainty calculated for listener-specific DTF sets (DTF varied, red circles). The abscissa is sorted by the ascending listener-specific uncertainty *U_k_*.

We used PEs and QEs predicted for all combinations of uncertainties and DTF sets, as shown in Figure 5. For each listener and each performance metric, two SDs were calculated: (1) as a function of the listener-specific DTF set *D_k_* for all available uncertainties, i.e., calculating the SDs across a column separately for each row; and (2) as a function of the listener-specific uncertainty *U_k_* for all available DTF sets, i.e. calculating the SD across a row separately for each column. Figure 7 shows these SDs as functions of the *k*-th listener, sorted by ascending listener-specific uncertainty. When *U_k_* was varied, the average SD across listeners was 4.4° ± 0.3° and 5.1% ± 0.4% for PE and QE, respectively. When the DTF set was varied, the average SD was 1.2° ± 0.1° and 1.9% ± 0.3% for PE and QE, respectively. On average, the factor uncertainty caused more than twice as much variability as the factor DTF set.

This analysis shows that while both listener-specific uncertainty and listener-specific DTF set were important for the accuracy in predicted localization performance, the uncertainty affected the performance much more than the DTF set. This indicates that the non-acoustic factor, uncertainty, contributes more than the acoustic factor, DTF set, to the localization performance. This is consistent with the observations of Andéol et al. (2013), where localization performance correlated with the detection thresholds for spectral modulation, but did not correlate with the prominence of the HRTF's spectral shape. The directional information captured by the spectral shape prominence corresponds to the acoustic factor in our study. The sensitivity to the spectral modulations represents the non-acoustic factor in our study. Even though the acoustic factor (DTF set) contributed to the localization performance of an individual listener, the differences *between* the listeners seem to be more determined by a non-acoustic factor (uncertainty).

Note that the separation of the sound localization process into acoustic and non-acoustic factors in our model assumes a perfect calibration of a listener to a DTF set. It should be considered, though, that listeners might actually be calibrated at different levels to their own DTFs. In such a case, the potentially different levels of calibration would be implicitly considered in the model by different uncertainties, confounding the interpretation of the relative contribution of the acoustic and non-acoustic factors. While the general capability to *re*-calibrate to a new DTF set has been investigated quite well (Hofman and van Opstal, 1998; Majdak et al., 2013), the level of calibration to the own DTF set has not been clarified yet.

## 4. Conclusions

In this study, a sound localization model predicting the localization performance in sagittal planes (Baumgartner et al., 2013) was applied to investigate the relative contributions of acoustic and non-acoustic factors to localization performance in the lateral range of ±30°. The acoustic factor was represented by the directional cues provided by the DTF sets of individual listeners. The non-acoustic factor was represented by the listener-specific uncertainty considered to describe processes related to the efficiency of processing the spectral cues. Listener-specific uncertainties were estimated in order to calibrate the model to the actual performance when localizing broadband noises with own ears. Then, predictions were calculated for the permutation of DTF sets and uncertainties across the listener group. Identical DTF sets were used for the incoming sound and the template set, which allowed to simulate the listeners being completely re-calibrated to the tested DTF sets, a condition nearly unachievable in psychoacoustic localization experiments.

Our results show that both the acoustic and non-acoustic factors affected the modeled localization performance. The non-acoustic factor had a strong effect on the predictions, and accounted very well for the differences between the individual listeners. In comparison, the acoustic factor had much less effect on the predictions. In an extreme case of using the same DTF set for modeling performance for all listeners, an acceptable prediction accuracy was still obtained.

Note that our investigation considered only targets positioned in sagittal planes of ±30° around the median plane. Even though we do not have evidence for contradicting conclusions for more lateral sagittal planes, one should be careful when applying our conclusions to more lateral targets. Further, the model assumes direction-static and stationary stimuli presented in the free field. In realistic listening situations, listeners can move their head, the acoustic signals are temporally fluctuating, and reverberation interacts with the direct sound.

An unexpected conclusion from our study is that, globally, i.e., on average across all considered directions, all the tested DTF sets encoded the directional information similarly well. It seems like listener-specific DTFs are not necessarily required for predicting the global listener-specific localization ability in terms of distinguishing between bad and good localizers. What seems to be required, however, is an accurate estimate of the listener-specific uncertainty. One could speculate that, given a potential relation between the uncertainty and a measure of spectral-shape sensitivity, in the future, the global listener-specific localization ability might be predictable by obtaining a measure of the listener-specific uncertainty in a non-spatial experimental task without any requirement of listener-specific localization responses.

### Conflict of interest statement

The authors declare that the research was conducted in the absence of any commercial or financial relationships that could be construed as a potential conflict of interest.

## References

[B1] Algazi V. R. Avendano C. Duda R. O. (2001). Elevation localization and head-related transfer function analysis at low frequencies. J. Acoust. Soc. Am. 109, 1110–1122 10.1121/1.1349185 11303925

[B2] Andéol G. Macpherson E. A. Sabin A. T. (2013). Sound localization in noise and sensitivity to spectral shape. Hear. Res. 304, 20–27 10.1016/j.heares.2013.06.001 23769958

[B3] Baumgartner R. Majdak P. Laback B. (2013). Assessment of sagittal-plane sound localization performance in spatial-audio applications, in The Technology of Binaural Listening, Modern Acoustics and Signal Processing, ed Blauert J. (Berlin; Heidelberg: Springer), 93–119

[B4] Baumgartner R. Majdak P. Laback B. (2014). Modeling Sound-Source Localization in Sagittal Planes for Human Listeners. Available online at: http://www.kfs.oeaw.ac.at/research/Baumgartner_et_al_2014.pdf (Last modified April 10, 2014). 10.1121/1.4887447 PMC4582445 25096113

[B5] Dau T. Püschel D. Kohlrausch A. (1996). A quantitative model of the “effective” signal processing in the auditory system. I. Model structure. J. Acoust. Soc. Am. 99, 3615–3622 10.1121/1.414959 8655793

[B6] Glasberg B. R. Moore B. C. J. (1990). Derivation of auditory filter shapes form notched-noise data. Hear. Res. 47, 103–138 10.1016/0378-5955(90)90170-T 2228789

[B7] Goupell M. J. Majdak P. Laback B. (2010). Median-plane sound localization as a function of the number of spectral channels using a channel vocoder. J. Acoust. Soc. Am. 127, 990–1001 10.1121/1.3283014 20136221 PMC3061453

[B8] Hofman P. M. van Opstal J. (1998). Spectro-temporal factors in two-dimensional human sound localization. J. Acoust. Soc. Am. 103, 2634–2648 10.1121/1.422784 9604358

[B9] Hofman P. M. van Riswick J. G. A. van Opstal J. (1998). Relearning sound localization with new ears. Nat. Neurosci. 1, 417–421 10.1038/1633 10196533

[B10] Langendijk E. H. A. Bronkhorst A. W. (2002). Contribution of spectral cues to human sound localization. J. Acoust. Soc. Am. 112, 1583–1596 10.1121/1.1501901 12398464

[B11] Macpherson E. A. Sabin A. T. (2007). Binaural weighting of monaural spectral cues for sound localization. J. Acoust. Soc. Am. 121, 3677–3688 10.1121/1.2722048 17552719

[B12] Majdak P. Balazs P. Laback B. (2007). Multiple exponential sweep method for fast measurement of head-related transfer functions. J. Audio. Eng. Soc. 55, 623–637

[B13] Majdak P. Goupell M. J. Laback B. (2010). 3-D localization of virtual sound sources: effects of visual environment, pointing method, and training. Atten. Percept. Psycho. 72, 454–469 10.3758/APP.72.2.454 20139459 PMC2885955

[B14] Majdak P. Walder T. Laback B. (2013). Effect of long-term training on sound localization performance with spectrally warped and band-limited head-related transfer functions. J. Acoust. Soc. Am. 134, 2148–2159 10.1121/1.4816543 23967945

[B15] Middlebrooks J. C. (1999a). Individual differences in external-ear transfer functions reduced by scaling in frequency. J. Acoust. Soc. Am. 106, 1480–1492 10.1121/1.427176 10489705

[B16] Middlebrooks J. C. (1999b). Virtual localization improved by scaling nonindividualized external-ear transfer functions in frequency. J. Acoust. Soc. Am. 106, 1493–1510 10.1121/1.427147 10489706

[B17] Møller H. Sørensen M. F. Hammershøi D. Jensen C. B. (1995). Head-related transfer functions of human subjects. J. Audio. Eng. Soc. 43, 300–321

[B18] Morimoto M. (2001). The contribution of two ears to the perception of vertical angle in sagittal planes. J. Acoust. Soc. Am. 109, 1596–1603 10.1121/1.1352084 11325130

[B19] Parseihian G. Katz B. F. G. (2012). Rapid head-related transfer function adaptation using a virtual auditory environment. J. Acoust. Soc. Am. 131, 2948–2957 10.1121/1.3687448 22501072

[B20] Patterson R. Nimmo-Smith I. Holdsworth J. Rice P. (1988). An Efficient Auditory Filterbank Based on the Gammatone Function. Cambridge: APU

[B21] Rakerd B. Hartmann W. M. McCaskey T. L. (1999). Identification and localization of sound sources in the median sagittal plane. J. Acoust. Soc. Am. 106, 2812–2820 10.1121/1.428129 10573897

[B22] Søndergaard P. Majdak P. (2013). The auditory modeling toolbox, in The Technology of Binaural Listening, Modern Acoustics and Signal Processing, ed Blauert J. (Berlin; Heidelberg: Springer), 33–56

[B23] van Wanrooij M. M. van Opstal J. (2005). Relearning sound localization with a new ear. J. Neurosci. 25, 5413–5424 10.1523/JNEUROSCI.0850-05.2005 15930391 PMC6724994

[B24] Vliegen J. van Opstal J. (2004). The influence of duration and level on human sound localization. J. Acoust. Soc. Am. 115, 1705–1703 10.1121/1.1687423 15101649

[B25] Wightman F. L. Kistler D. J. (1997). Monaural sound localization revisited. J. Acoust. Soc. Am. 101, 1050–1063 10.1121/1.418029 9035397

[B26] Zahorik P. Bangayan P. Sundareswaran V. Wang K. Tam C. (2006). Perceptual recalibration in human sound localization: learning to remediate front-back reversals. J. Acoust. Soc. Am. 120, 343–359 10.1121/1.2208429 16875231

[B27] Zakarauskas P. Cynader M. S. (1993). A computational theory of spectral cue localization. J. Acoust. Soc. Am. 94, 1323–1331 10.1121/1.408160

[B28] Zhang P. X. Hartmann W. M. (2010). On the ability of human listeners to distinguish between front and back. Hear. Res. 260, 30–46 10.1016/j.heares.2009.11.001 19900525 PMC2814998

